# Application of Integrated Behavioral Model (IBM) to measure intention to get early screening and treatment of Sexually Transmitted Infections (STIs) among HIV at- risk sub-populations in Ethiopia

**DOI:** 10.4314/ahs.v21i2.8

**Published:** 2021-06

**Authors:** Wondwossen Asefa Alemayehu, Jeanitte Maritz, Lizeth Roets

**Affiliations:** University of South Africa

**Keywords:** Behavior, integrated behavioral model, sexually transmitted infections, human immunodeficiency virus

## Abstract

**Background:**

Sexually Transmitted Infections (STIs) increase the risk of contracting Human Immunodeficiency Virus (HIV). Hence, early screening and treatment of STIs as a behavioral practice will reduce the odds of HIV infection among at risk and vulnerable sub-populations. To that end, HIV prevention strategies need to design evidence-based interventions using behavioral models or theories to help at-risk individuals adopt early screening and treatment of STI as preventive health behavior. In this study, commercial sex workers were considered as HIV at-risk sub-populations.

**Objective:**

Measuring to what extent that Integrated Behavioral Model constructs explain individuals' intention to practice early screening and treatment of sexually transmitted infections as healthy behavior of interest in HIV prevention.

**Design:**

Integrated Behavioral Model (IBM) measurement survey was conducted using Respondent Driven Sampling (RDS) in six towns located in the main transport corridors of Ethiopia. Respondents' answers to model construct-based questions and intention to practice the health behavior of interest were measured using Likert Scale. Analysis was done to assess the correlation and level of association of model construct-based questions with intention to practice the preventive health behavior.

**Results:**

Respondents' attitude explained 32%, perceived control 2%, normative influence 21%, and self-efficacy 53 % of their intention to get early screening and treatment of sexually transmitted infections.

**Conclusion:**

Self-efficacy explained the variability of respondents' intention to get early screening and treatment of STIs most, while perceived control was the least. Hence, HIV prevention behavioral interventions targeting early screening and treatment of STIs should give high emphasis to self-efficacy.

## Introduction

Acquisition and transmission of HIV infection has been associated with bacterial and viral Sexually Transmitted Infections (STIs). This was first demonstrated in case series and retrospective studies that showed an association between previous sexually transmitted infections and human immunodeficiency virus 1,2. From susceptibility to infection and infectivity standpoints, there is high level of association between genital ulcerations and HIV transmission. Infections with microorganisms such as *Treponema pallidum, Haemophilus ducreyi*^3^. Fauci and Lane^3^ further states etiologic factors responsible for non-ulcerative inflammatory STIs such as those caused by *Neisseria gonorrhoeae, Chlamydia trachomatis*, and *Trichomonas vaginalis* are associated with an increased risk of transmission of HIV infection. Studies in Southern Africa showed more than 40% decreases of HIV incidence by controlling STIs even though it is dependent on the length of screening and treatment intervention and other factors^4^. Hence, early screening and treatment of STIs among at risk population groups (e.g commercial sex workers) has paramount importance to reduce their susceptibility and odds of contracting HIV. The Most at Risk Population (MARPs) survey in Ethiopia showed the highest prevalence of HIV infection (23%) among self-identified commercial sex workers 5 . Studies also showed higher STI prevalence (20.6%–47.9%) among commercial sex workers in different parts of Ethiopia 6,7. Hence, in this study, commercial sex workers' behavior to get early screening and treatment of STIs was considered as important HIV preventive health behavior of interest targeting most atrisk sub-population group.

Health behavior and its determinants are broadly defined as “those personal attributes such as beliefs, expectations, motives, values, perceptions, and other cognitive elements; personality characteristics, including affective and emotional states and traits; and overt behavior patterns, actions, and habits that relate to health maintenance, to health restoration, and to health improvement”^8^. A mounting body of evidence suggests that behavioral interventions developed using explicit theoretical foundation or models are more effective than those lacking a theoretical base 9. Theory- or model-based health behavior intervention programs help to better understand the mechanism of behavior change and guide the intervention for improved impact 10. Behavior interventions informed by theories and models are more effective as they include essential factors necessary for behavior change 11. A review of online health behavior interventions demonstrated that theory-based interventions have been more successful in prompting behavior change than those without theory 12. Besides its effectiveness, advocates for theory-based intervention as theoretical understanding of behavioral intervention creates enabling environment for adaptation of interventions into other settings 13. To that end, measuring the application of behavioral theories/models is important step to inform theory/model based behavioral interventions 14. Hence, this study aims to assess the application of Integrated Behavioral Model (IBM) among HIV at-risk sub-populations by measuring the model constructs (Experiential Attitude, Instrumental Attitude, Normative Influence, Perceived Control, and Self-efficacy) ability to explain the study subjects' intention to adopt the health behavior of interest (early screening and treatment of STIs). As early screening and treatment of STIs is important HIV prevention behavior and practice among at risk sub-population like sex workers. Integrated Behavioral Model (IBM) was selected for this study since it is more comprehensive conceptual framework that puts intention to perform the behavior at the center and account for different health behaviors in different populations and settings^15^, ^16^,^17^. Moreover, studies showed that Integrated Behavioral Model has been useful in terms of predicting and guiding HIV/AIDS related health behavioral studies and interventions including condom use, early sexual debut, awareness, and HIV testing that are quite related with the objectives of this study 18.

## Objective

In this study, Integrated Behavioral Model (IBM) based behavioral constructs were used to measure the magnitude each behavioral model construct explains intention to practice the desired health behavior. Early screening and treatment of STIs being the desired health behavior to prevent HIV infection among commercial sex workers. Such kind of model measurement is helpful to determine which construct best explain intention to perform the desired health behavior, and the level of emphasis that should be given to different constructs on a given HIV preventive behavioral intervention. The model measurement focuses on “intention” since it is the best predictor and centerpiece of the behavioral practice in the integrated behavioral model 15.

## Methods and materials

Integrated Behavioral Model (IBM) measurement survey was conducted in six towns (Dessie, Bahir Dar, Dire Dawa, Hawassa, Jimma and Nekemet) located along the six major transport corridors of Ethiopia. The survey was conducted among self-acknowledged commercial sex workers that were identified as highly vulnerable and most at- risk for HIV and other STIs according to studies in Ethiopia 5 . The very first step in the IBM model measurement was to discover salient issues through open-ended opinion elicitation (elicitation study). Elicitation is very critical step in the application of IBM constructs^15^. The elicitation phase was conducted to identify relevant behavioral outcomes, referents, and environmental facilitators and barriers for the health behavior and population under investigation. According to Montaño and Kasprzyk^15^ the minimum number of opinion elicitation interviews are^15^. In this study, a total of 20 opinion elicitation interviews were conducted until saturation has reached and to insure maximum variation. This was meant to identify salient issues about the behavioral factor of interest (early screening and treatment of STIs). The modal response about early screening and treatment of STIs was affirmative. Peer group members and health workers' normative influence over commercial sex workers behavior to seek early screening and treatment of STIs stood out remarkably. The peer education and peer support program in the community was mentioned as a major enabler followed by the availability of free screening and treatment services of STIs in public and selected private health care facilities. The elicitation results were content analyzed to inform the model measurement survey. Respondent Driven Sampling (RDS) was used to select and interview model measurement survey participants in the six study towns. Respondent Driven Sampling has gained enormous popularity as it minimizes bias and increases representativeness that has been an intractable dilemma in studying highly vulnerable and hard to reach population groups^19^. By limiting number of recruitments per seeds and introducing multiple waves of recruitment phases, this sampling technique helps reducing recruitment bias, high homophily and deeper penetration of the target population for representativeness purpose 20. The first five “seeds” were randomly selected from existing social networks of commercial sex workers participating in HIV prevention projects in the six towns. In the second wave of recruitment, two invitations were handed-out for the first five “seeds” to get the next ten self-identified commercial sex workers in their network (peer group). Subsequent waves of recruitments have continued likewise till the maximum number of respondents were reached. Single population proportion sample size determination formula has been used to determine the number of model measurement survey participants. With 95% confidence interval and 5% margin of error, 50% P was applied to get the maximum sample size of 385. Adjusting the sample size for 5% loss to network referrals and a minimum of 3–4 waves of requirement to get deeper penetration, the total sample size for the model measurement survey was 405. Integrated Behavioral Model (IBM) construct-based Likert Scale was used to design the model measurement questioner. The model measurement survey questioner was prepared in English and translated to Amharic (local language). The Amharic version has been translated back to English independently by another person with good Englih proficiency and familiar to data collection instruments to see possible inconsistencies and prevent meaning loss through the course of translation. Scaled bipolar and unipolar survey questioner was developed to measure the constructs of the behavioral model of interest. Respondents' answers to model construct-based questions and intention to practice the health behavior of interest were measured using seven scale Likert Scale. Further analysis was done to compute the correlation coefficients between the responses to model construct based questions and intention to practice the desired health behavior. Pearson's correlation coefficient (r) and (R2) were calculated to determine the level of correlation and extent of prediction respectively. Pearson's correlation coefficient was preferred as it measures the statistical relationship between two continuous variables and it gives information about the magnitude of the correlation, as well as the direction of the relationship^21^. The degree of correlation between the predictor and outcome variables was defined as follow based on the value of “r” 21,22:

- Perfect: If the value is near ± 1
- High degree: If the coefficient value lies between ± 0.50 and ± 1
- Moderate degree: If the value lies between ± 0.30 and ± 0.49
- Low degree: When the value lies below + .29
- No correlation: When the value is zero

The rating of model construct-based question's response and intention to practice the health behavior of interest was further analyzed using liner regression model to determine the extent to which the model constructs predict intention. Hence, respondents rating of “intention” to practice the health behavior of interest (early screening and treatment of STIs) was considered as dependent variable while respondents rating for their response under the five IBM construct questions were considered as independent (predictor) variable in the regression model. The five IBM constructs under which the independent variables were created are: Experiential Attitude, Instrumental Attitude, Normative Influence, Perceived Control, and Self-efficacy.

## Results and discussions

Three hundred ninety respondents participated in the study, which was 96% response rate. The mean and median age of respondents was 25 and 24 years respectively with standard deviation of ± 4.4 and range of 24 years. The average year of schooling by the study participants was six years and the 75th percentile was eight years of schooling. Most of the participants (63%) were single followed by divorced 19%, married 11% and widowed 7%.

### Correlation of salient issues with intention to practice the health behavior of interest

#### Attitude (Experiential and Instrumental)

Attitude refers to an individual's overall perception of favorableness or un-favorableness towards a behavior comprised of affective and cognitive dimensions. Experiential attitude (or affect) is the individual's emotional response to the idea of performing the behavior while Instrumental attitude (or cognitive) is determined by beliefs about outcomes of behavior 8. Because of the close correlation of the two constructs, commercial sex workers experiential and instrumental attitude regarding STIs as defined by their overall perception of favorableness or un-favorableness to get early screening and treatment services of sexually transmitted infections were combined in the correlation analysis. This reflects on commercial sex workers feeling and emotional reaction about performing STI screening and treatment earlier. Table 1 depicts the correlation of sex workers attitude to their intention of getting early screening and treatment of STIs.

**Table 1:** The correlation between respondents' attitude and their intention to get early screening and treatment services of Sexually Transmitted Infection (STIs)

Intention to get early screening and treatment of STIs	r	Sig. (2- tailed)	N
⇒ Concern about complications and serious illness	.542**	.000	390
⇒ Senses of relief	.399**	.000	390
⇒ Preventing yourself and others from infections	.293**	.000	390

** Correlation is significant at the 0.01 level (2-tailed)

Perceived complications, sense of relief, and preventing oneself or others from infection were correlated with commercial sex workers' intention to get early screening and treatment services of sexually transmitted infections at statistically significant margin.

#### Perceived control

This was commercial sex workers self-understood level of control to practice early screening and treatment of sexually transmitted infections.

As illustrated above (Table 2), availability of free STI screening and treatment services and peer education program were correlated with commercial sex workers' intention to get early screening and treatment services of STIs at statistically significant margin.

**Table 2:** The correlation between respondents' perceived control and their intention to get early screening and treatment services of Sexually Transmitted Infection (STIs)

Intention to get early screening and treatment of STIs	r	Sig. (2- tailed)	N
⇒ Availability of free STI screening and treatment services	.145**	.004	390
⇒ Availability of peer education program	.135**	.008	390
⇒ Friendly service providers or good reception in the clinics	.028	.588	390
⇒ Friends and colleagues discouragement	-.036	.475	390

** Correlation is significant at the 0.01 level (2-tailed)

#### Normative influence

Normative influence was the community members' beliefs and expectations regarding early screening and treatment of sexually transmitted infections and how that affects commercial sex workers to perform this healthy behavior.

Table 3 shows the correlation of normative influencers with sex workers intention to get early screening and treatment of STIs. Thus, peer group members and health care workers influence in the community showed strong correlation with commercial sex workers' intention to get early screening and treatment of STIs at statistically significant margin.

**Table 3:** The correlation between normative influence and respondents' intention to get early screening and treatment services of Sexually Transmitted Infection (STIs)

Intention to get early screening and treatment of STIs	r	Sig. (2- tailed)	N
⇒ Peer educators and peer group members	.389**	.000	390
⇒ Health workers in the community and clinics	.355**	.000	390

** Correlation is significant at the 0.01 level (2-tailed)

#### Self-efficacy

This was commercial sex workers' skill and capability to effectively practice early screening and treatment of sexually transmitted infections as a tool for HIV prevention. Table 4 shows the correlation of self-efficacy with sex workers intention to get early screening and treatment of STIs.

**Table 4:** The correlation between respondents' self-efficacy and their intention to get early screening and treatment services of Sexually Transmitted Infection (STIs)

Intention to get early screening and treatment of STIs	r	Sig. (2- tailed)	N
⇒ Certainty to practice early screening and treatment of STIs	.657**	.000	390
⇒ Certainty that peer education program helps to overcome barriers to get early screening and treatment of STIs	.684**	.000	390
⇒ Certainty that free STI screening and treatment service helps to overcome barrier to get the service	.277**	.000	390

** Correlation is significant at the 0.01 level (2-tailed)

Self-reported certainty of commercial sex workers to practice STI screening and treatment, peer-education program, and free STI screening and treatment services demonstrated strong correlation with commercial sex workers' intention of using early screening and treatment services of STIs at statistically significant margin.

#### Measurement levels

To measure the extent how the Integrated Behavioral Model (IBM) constructs explain respondents' intention to get early screening and treatment of STI, linear regression model was used. The seven-scale Likert measurement of respondents' intention to get early screening and treatment of STI was entered into the model as dependent variable. All the other STI related behavioral measurement questions under the five IBM constructs were entered into the model as predictor (independent variables). The model output was interpreted by R-squared (R2) which was a statistical measure of how close the data were to the fitted regression line which was the percentage of the response variable variation that was explained by a linear model. All integrated behavioral model constructs combined explained 61% of commercial sex workers' intention to practice early screening and treatment of STIs as depicted in the underneath model summary table (Table 5).

**Table 5:** Model measurement summary table early screening and treatment of STI and all IBM constructs

Model Summary				
Model	R	R Square	Adjusted R Square	Std. Error of the Estimate
1	.791a	.625	.613	.626

#### Attitudes (Experiential and Instrumental)

Respondents' attitude (experiential and instrumental), explained 32% of their intention to get early screening and treatment of sexually transmitted infections as depicted in the adjusted R2 of the following model summary (Table 6).

**Table 6:** Model measurement summary table early screening and treatment of STI and attitude

Model Summary				
Model	R	R Square	Adjusted R Square	Std. Error of the Estimate
1	.571^a^	.327	.321	.829

a Predictors: (Constant), Early screening and treatment of STIs mean preventing yourself and others from infections, Early screening and treatment f STIs will give you senses of relief, Early scr eening and treatment of STIs mean you do not have to worry about complications and serious illness

#### Perceived control

The other IBM construct measured in the regression model was respondents' perceived control to get early screening and treatment of sexually transmitted infections. As shown in the below model summary (Table 7), respondents' perceived control was the list behavioral model construct that explains their intention to get early screening and treatment of STIs with 3% and 2% unadjusted and adjusted R2 values respectively.

**Table 7:** Model measurement summary table early screening and treatment of STI and perceived control

Model Summary				
Model	R	R Square	Adjusted R Square	Std. Error of the Estimate
1	.172^a^	.030	.020	.997

a Predictors: (Constant), Friends and colleagues discouragement will make your early screening and treatment of STI decision harder, How easier does free STI screening and treatment services makes you to use the service, Friendly service providers or good reception in the clinics will make your early screening and treatment of STI practice easier, How easier does peer education program makes your early screening and treatment practice.

#### Normative influence

Respondents' normative influence was the fourth behavioral model construct that was measured in the linear regression model to assess the extent how the construct explains study subjects' intention to get early screening and treatment of STIs. With 22% of unadjusted and 21% of adjusted R2 values respectively (Table 8), it is the third important IBM model construct that explains respondents' intention to get early screening and treatment service of sexually transmitted infections.

**Table 8:** Model measurement summary table early screening and treatment of STI and normative influence

Model Summary				
Model	R	R Square	Adjusted R Square	Std. Error of the Estimate
1	.466^a^	.217	.213	.893

a Predictors: (Constant), When it comes to early screening and treatment of STIs, how helpful are health workers in the community and clinics., When it comes to early screening and treatment of STIs, how helpful are peer educators and peer group members.

#### Self-efficacy

Respondents' self-efficacy was the fifth IBM construct that was measured in terms of its influence to explain respondents' intention to get early screening and treatment of STIs. As shown in the underneath model summary (Table 9), self-efficacy explains 54% (unadjusted) and 53% (adjusted) of respondents' intention to get early screening and treatment of sexually transmitted infections. This demonstrates that, self-efficacy is the 299 highest behavioral model construct that explains the variability of respondents' intention to get early screening and treatment of STIs.

**Table 9:** Model measurement summary table early screening and treatment of STI and self-efficacy

Model Summary				
Model	R	R Square	Adjusted R Square	Std. Error of the Estimate
1	.732^a^	.536	.533	.688

a Predictors: (Constant), How certain are you that free STI screening and treatment service helps you to overcome barrier to get the service, How certain are you that peer education program helps you to overcome any barriers to get early screening and treatment of STIs, How certain are you to practice early screening and treatment of STIs

## Conclusions and recommendations

Peer group members and health care workers influence in the community have showed strong correlation with commercial sex workers' intention to get early screening and treatment of STIs at statistically significant margin. Hence, HIV prevention behavioral intervention programs targeting commercial sex workers to adopt early screening and treatment of STIs as healthy behavior may use their peers and health care workers to educate and better influence intention to perform this preventive health behavior.

Integrated Behavioral Model (IBM) has explained 61% of commercial sex workers' intention to practice early screening and treatment of STIs. With adjusted R2 =0.53, self-efficacy was the most important behavioral model construct in terms of explaining the variability of respondents' intention to get early screening and treatment of STIs. Thus, IBM based HIV prevention behavioral intervention programs targeting at risk sub-populations (e.g. commercial sex workers) should give more emphasis (time and focus) on message contents to influence their self-efficacy to get STI screening and treatment earlier.

Perceived control of commercial sex workers has the least effect (adjusted R2=0.02) in terms of explaining their intention to perform early screening and treatment of STIs. Hence, behavioral intervention strategies focused on early screening and treatment of STIs should give very little attention to influence commercial sex workers' perceived control.

HIV prevention behavioral interventions focusing on commercial sex workers' attitudes and normative influence to adopt early screening and treatment of STIs deemed to explain their intention to practice the health behavior of interest modestly (32% and 21% respectively). Thus, proportional level of behavioral intervention emphasis is recommended for IBM based HIV prevention program targeting commercial sex workers.

## Abbreviations

AIDS: Acquired Immuno Deficiency Syndrome
EPHI: Ethiopian Public Health Institute
HIV: Human Immunodeficiency Virus
IBM: Integrated Behavioral Model
IRB: Institutional Review Board
RDS: Respondent Driven Sampling
STI: Sexually Transmitted Infection

## Declarations

### Availability of data and material

The raw data supporting the results reported in this article can be shared to interested individuals or institutions based on written request through the corresponding author.
